# Beyond Antibiotics: What the Future Holds

**DOI:** 10.3390/antibiotics13100919

**Published:** 2024-09-25

**Authors:** Payam Benyamini

**Affiliations:** Department of Health Sciences at Extension, University of California Los Angeles, 1145 Gayley Ave., Los Angeles, CA 90024, USA; payamb@ucla.edu

**Keywords:** phage therapy, pilicides, quorum sensing and biofilm inhibitors, toxoids, microfloral transplants, type III secretion system inhibitors

## Abstract

The prevalence of multidrug resistance (MDR) and stagnant drug-development pipelines have led to the rapid rise of hard-to-treat antibiotic-resistant bacterial infections. These infectious diseases are no longer just nosocomial but are also becoming community-acquired. The spread of MDR has reached a crisis level that needs immediate attention. The landmark O’Neill report projects that by 2050, mortality rates associated with MDR bacterial infections will surpass mortality rates associated with individuals afflicted with cancer. Since conventional antimicrobials are no longer very reliable, it is of great importance to investigate different strategies to combat these life-threatening infectious diseases. Here, we provide an overview of recent advances in viable alternative treatment strategies mainly targeting a pathogen’s virulence capability rather than viability. Topics include small molecule and immune inhibition of virulence factors, quorum sensing (QS) quenching, inhibition of biofilm development, bacteriophage-mediated therapy, and manipulation of an individual’s macroflora to combat MDR bacterial infections.

## 1. Introduction

Prior to the beginning of the 20th century, the average life expectancy of both men and women in the industrialized world was ~47 years of age [1]. At the time, infectious diseases such as cholera, pneumonia, typhus, plague, tuberculosis, etc., ran rampant worldwide and led to very high morbidity and mortality rates. For example, in the early 1900s, the mortality rate for endocarditis was 100%, >95% for meningitis, 30% for pneumonia, and 10% for serious skin infections [1,2].

In 1893, the Italian microbiologist Bartolomeo Gosio was the first to discover an antibiotic derived from *Penicillium glaucum*, known as mycophenolic acid, which was shown to inhibit the growth of *Bacillus anthracis*. Following this initial discovery, Paul Ehrlich and his colleagues identified the synthetic organarsenic compound Salvarsan in 1909, the first synthetic arsenic-derived antibiotic with efficacy against *Treponema pallidum*. Due to side effects associated with Salvarsan, Neosalvarsan was subsequently introduced in 1913 for the treatment of syphilis [3]. The turning point in our ability to fight such devastating bacterial infections on a broader scale came with the accidental discovery of penicillin in 1928 [4]. At the time, penicillin was the first ß-lactam antibiotic capable of inhibiting the growth of the superbug *Staphylococcus aureus*. This marked the beginning of the antibiotic revolution. Since then, antibiotics have served as the cornerstone of modern medicine and have proven to be indispensable in our fight against deadly bacterial pathogens [5,6]. There are two general classes of antibiotics: bactericidal drugs, which display greater efficacy by directly killing the pathogen, and bacteriostatic drugs, which inhibit growth as long as the drug persists in the patient’s system at an adequate concentration [3]. However, decades of selective pressures posed by unregulated industrial overuse, as well as inappropriate or misuse of antibiotics, has led to the vigorous rise of a plethora of multidrug-resistant (MDR) bacterial pathogens [7,8], thereby presenting a direct threat to the achievements of the antibiotic renaissance era.

A pathogen’s ability to develop resistance against antibiotics is a widespread, naturally occurring phenomenon that predates its discovery and is very difficult to mitigate [9,10]. Acquisition of drug resistance is a natural coping mechanism employed by all bacteria to provide a competitive advantage. Resistance results from random mutations in microbial genomes, genetic drift among microbial populations, and horizontal gene transfer [11,12,13]. However, it should be stressed that a majority of worldwide cases associated with the rapidly growing emergence of drug resistance are prompted by behavioral and socioeconomic factors [14,15,16]. These accelerating factors include overuse, misuse, poverty, minimal access to quality healthcare and medicines, international travel, inadequate implementation of infection control practices, overcrowding, scarcity of resources, and, in some cases, technological advancement [14,15,17,18,19,20]. Unfortunately, the absence of innovation and the lack of relevant research focused on identifying and developing new classes of antibiotics has undermined our ability to treat general infections in clinical practice.

With the global rise in antibiotic resistance, the World Health Organization (WHO) states that international antibiotic drug-development pipelines have become stagnant and do not address the mounting threat of the rapid rise of MDR bacterial pathogens [21,22]. As of September 2023, the antibiotic pipeline includes 68 new chemical entities, of which 18 fulfill the WHO standard of novelty [23,24,25]. Twenty-four are known to target the WHO priority pathogens, followed by twelve targeting *Mycobacterium tuberculosis* and five targeting *Clostridium difficile*. Out of eleven novel antibiotics approved for the clinical setting, 80% belong to the existing classes [23,24,26]. Only a few antibiotics approved over the past 40 years represent new compound classes, and the majority are derived from known chemical entities. Of these nine new antibiotics, only five can target Carbapenem-resistant Enterobacteriaceae (CRE), and only one can penetrate the outer membrane of GNP and show activity against CRE, Carbapenem-resistant *Acinetobacter baumannii* (CRAB), and carbapenem-resistant *Pseudomonas aeruginosa* (CRPA). To date, only a few drug leads are in the pipeline with the ability to target critically important Gram-negative bacteria (GNB) and stop the spread of MDR clinical isolates [3,21,27].

Currently, the rate of new drug-class entry into the market is very slow [28,29]. This is due to various factors, such as many drug leads not making it past the early stages of development due to toxicity and off-target effects [30,31,32]. In many cases, big pharma companies are disinterested in antibiotics owing to their limited lifespan, which results in a poor return on investment [33,34]. At this pace, many pathogens are inherently going to become more drug-resistant, while fewer antimicrobials will make it to market. The landmark O’Neill report pertaining to the global problem of antimicrobial resistance estimates that by 2050, the mortality rate associated with bacterial infections will surpass cancer [35,36,37].

We have learned a great deal about antibiotics in the past 85 years. Understanding the mechanism of action underlying each class of antibiotic is centered on the cellular process that is inhibited by the interplay between a drug and its target. Antibiotics can be classified as either being bacteriostatic (inhibit cell growth) or bactericidal (cause cell death) [38]. The most current classes of antibiotics specifically target one of multiple cell functions, such as DNA, RNA, and protein synthesis or cell-wall assembly, as well as membrane-targeting agents and anti-metabolites [39,40]. Antibiotic-associated cell death is a complicated process that involves physical drug–target interaction. This interplay results in physiochemical and ultrastructural changes in the bacterium that lead to cell death. Antibiotics display a variety of specific mechanisms of action, such as (A) topoisomerase inhibitors, which cause cell death by inducing double-stranded DNA breaks and arresting DNA replication, (B) protein synthesis inhibitors that bind directly to either ribosomal subunits and cause protein mistranslation, (C) cell-wall synthesis inhibitors that compromise the structural integrity of the cell-wall, (D) metabolic inhibitors that induce stasis or cell death, and lastly, (E) membrane targeting antimicrobials that disrupt cell membrane integrity. However, the rapid emergence of MDR is threatening this therapeutic accomplishment [40].

When introduced into the clinic, the efficacy of antibiotics is high; however, small populations of pathogenic bacteria gradually develop resistance and continue to persist in a host. Bacterial pathogens develop resistance via various mechanisms that include enzymatic hydrolysis, overexpression of efflux pumps and drug targets, downregulation of porins, and horizontal acquisition of antibiotic resistance genes. Published reports reveal that MDR is responsible for approximately 700,000 deaths annually, and if left unresolved, it’s predicted to reach 20 million by 2050 [3].

In addition to the rapid rise in MDR, non-antibacterial side effects associated with antibiotics exist due to the endosymbiotic origins of bacteria and mitochondria [41,42]. For example, published reports reveal that various antibiotics such as ciprofloxacin, tetracycline, quinolones, aminoglycosides, ß-lactams, etc., cause mitochondrial dysfunction, such as targeting of mitochondrial DNA replication, protein synthesis, metabolism, membrane potential, the electron transport chain and production of reactive oxygen species even at clinically relevant doses [43,44,45,46,47,48], thereby further complicating the use of antibiotics in clinical settings.

The clinical landscape of antimicrobials is changing due to the limited lifespan of antibiotics, the inherent emergence of drug resistance, and observed toxicity in eukaryotic cells [43,49]. Alternative non-traditional strategies are required to approach such a threat. There are three key aspects to the development of successful anti-infective therapies: (A) Identification of novel classes of antimicrobial agents that inhibit existing targets but with a different mechanism of action; (B) Identification of new therapeutic targets; and (C) development of antibiotic-independent approaches that minimize the emergence of drug-resistance.

Virulence factors enhance a bacterium’s ability to colonize and adapt to its host. These factors can either be membrane-bound, cytosolic effectors, or secreted into the extracellular milieu [50,51]. Membrane-bound virulence factors enable physical interaction between the host and the pathogen for colonization or biofilm formation [52,53]. Species-specific cytosolic effector proteins are directly injected into host cells by means of different types of secretion systems, specifically, the type III secretion system (T3SS) [54,55,56,57,58]. These injected effectors commandeer host cells to regulate cytoskeletal organization, cell proliferation, survival, stress responses, and physiological capabilities [57,59,60]. Secreted virulence factors shape the overall bacterial armory by enabling the pathogen to overcome host immune defenses, invade deeper tissues, and disseminate to distal anatomical sites [50]. In many cases, virulence factors work synergistically to kill host cells at different stages of disease [61,62,63]. It should be noted that in many cases, the targeting of virulence factors does not kill the pathogen directly; rather, it significantly limits virulence, making it possible to introduce bactericidal combination therapy at lower concentrations.

In the following review, we will highlight recent advances in the fields of immune and small molecule therapies targeting specific virulence factors, such as type III secretion systems, quorum sensing (QS) capabilities, biofilm formation, targeting of toxins for vaccine purposes, bacteriophage therapy and manipulation of an individual’s macroflora as viable treatment options to combat MDR bacterial infections.

## 2. Targeting Type III Secretion Systems (T3SS)

As part of their mechanism of pathogenesis, numerous GNB deploy membrane-spanning and soluble multi-protein structures known as T3SS. These secretion systems inject effector proteins through eukaryotic cell membranes into the cytosol of host cells [54]. The effectors take control of essential host cell functions necessary for survival. Given their role in the pathogenic capacity of various human pathogens such as *Salmonella* spp., *Shigella* spp., *Yersinia* spp., pathogenic *Escherichia coli*, *Vibrio* spp., *Pseudomonas* spp., *Chlamydia* spp., T3SS serve as ideal target candidates for the discovery and development of novel antimicrobials [64,65,66,67,68,69,70,71,72]. It is reported that defects in T3SS assembly render a pathogen avirulent, and the advantage of targeting secretion systems is that they are typically associated with only pathogenic bacteria [72]. For example, prior to phagocytosis, *P. aeruginosa* uses its T3SS to inject effector proteins that attenuate neutrophils by subverting an increased production of antimicrobial peptides (AMPs) and the generation of reactive oxygen species [73]. So, targeting this system does not affect the vast commensal population, thereby decreasing the likelihood that a resistant strain originates from the host’s own microflora, as is the case with the administration of conventional antibiotics.

**Figure 1:**
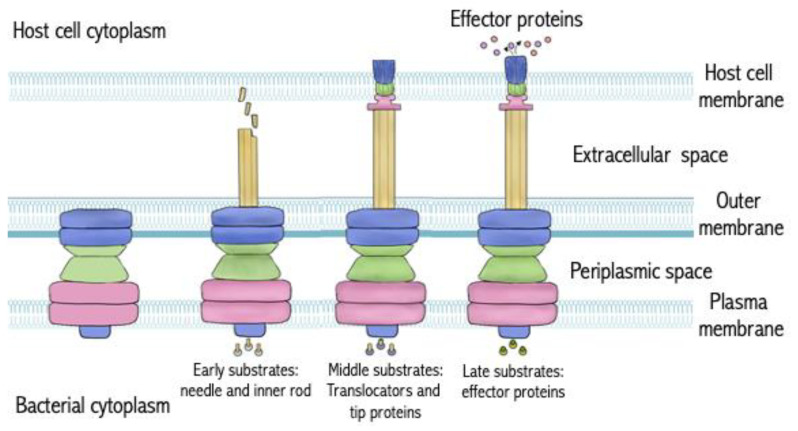
Type III secretion systems comprise two dozen different oligomers that adopt a syringe-like structural configuration. The T3SS is made of a cytosolic sorting platform, a hollow multiring basal structure that spans both bacterial membranes, and an extracellular needle-like appendage that punctures the host plasma membrane for the translocation of effector proteins into the host cytoplasm.

In contrast to effector proteins that are species-specific, T3SS are encoded by a set of evolutionarily conserved homologous genes expressed by numerous GNB species [74]. These systems comprise different subunits that assemble into nanoscale syringe-like structures that span the inner membrane, periplasm, and outer membrane of GNB and penetrate through eukaryotic cell envelopes to deliver effector proteins into the cytosol. The core secretion machinery is composed of two dozen different oligomers that adopt a general structural configuration, including a cytosolic sorting platform, a hollow multiring basal structure that spans both bacterial membranes, and an extracellular needle-like appendage that encloses the export apparatus comprising inner membrane proteins [75]. An ATPase is responsible for providing the necessary energy to unfold and introduce the effector proteins from the cytoplasm into the secretion system [76,77]. The needle serves as a scaffold for the assembly of a hollow extracellular channel responsible for forming a pore in host cell membranes and injecting the partially unfolded effector protein into the host cytoplasm (Figure 1) [55,74,78,79,80,81,82,83]. Interestingly, numerous GNB species display highly conserved T3SS protein structures, so multi-species treatment modalities can be possible [84,85].

To date, several new classes of small molecule inhibitors have been identified that block the secretion of effector proteins from enteropathogenic *E. coli* (EPEC). For example, a study performed by Mühlen et al. screening two natural and four chemical compound libraries with a total of 13,360 substances identified 50 small molecules capable of reducing translocation efficiency by 50 to 75%, while another 27 interfered with effector translocation by more than 75% compared to untreated controls. Additionally, 24 compounds showed a reduction of 100% [67]. The latter corresponded to known or published antimicrobials, including chloramphenicol, rifampin, thuggacin, and tartrolon, as well as derivatives of myxovirescin, myxovalargin, and sorangicin. Furthermore, an assessment of the effects of primary hits of small molecules on bacterial growth and cell viability revealed a dose-response inhibitory effect associated with 56 molecules that were never published. Of these, 35 compounds were confirmed to inhibit translocation, and eight showed a reduction of EPEC growth by over 75% at the highest tested concentration of 50 µM [67].

High-throughput screening of two different small molecule libraries (SPECS and Var) identified compounds S3 (1,2,4-triazine-5-one) and S4 (p-methoxy-hydrocinnamamide) from SPECS and S6 (3-chloroquinoxalin-2(1H)-one 4-oxide) from the Var showed inhibitory action against effector protein translocation without cytotoxicity to eukaryotic cells. The concentration of these compounds with maximal inhibition of effector translocation without adverse effects on eukaryotic cells was 50 µM for S3 and S4 and 25 µM for S6. It is established that hemolysis of erythrocytes is T3SS-dependent and has been attributed to the formation of the T3SS translocon pore in erythrocyte membranes. Assessment of hemolytic activity of sheep-derived erythrocytes revealed that S3 had no effect on hemolysis, whereas S4 and S6 significantly inhibited hemolysis by 20% and 10%, respectively, indicating that S3 mechanism of action does not interfere with the assembly and translocon pore formation by the T3SS [67]. Several studies show compounds that belong to a class of acylated hydrazones of different salicylaldehydes target the T3SS system of *Yersinia*, *Salmonella*, *Chlamydia*, *E. coli*, and *Shigella* [64,68,86,87,88,89,90].

A study published by Felise et al. (2008) indicates that the compound 2-imino-5-arylidene thiazolidinone also inhibited T3SS-dependent functions [91]. Wolf et al. found that another compound known as N′-(3,5-dibromo-2-hydroxybenzylidene)-4-nitrobenzo-hydrazide had inhibitory activity against *Yersinia* T3SS [92]. Using a luciferase reporter gene system in *Yersinia pseudotuberculosis*, Kauppi et al. (2003) screened approximately 9400 small molecules and identified several compounds that target the T3SS machinery at different levels [93]. High-throughput screening of 70,966 by Pan et al. identified eight compounds with T3SS inhibitory activity. Four of the eight compounds effectively inhibited effector proteins at low µM concentrations [94]. Studies performed by Kimura et al. (2010) revealed that linear polyketides obtained from the culture broths of *Streptomyces* spp. also protect against *Citrobacter rodentium* in vivo [95]. Additionally, Larzabal et al. (2010) also revealed that coiled-coil peptides can prevent the assembly of T3SS, rendering it non-functional [96]. Natural occurring products such as caminosides, aurodox, piericidin A, cytosporone B, cuadinomines, butyric acid, fusaric acid, (−)-hopeaphenol, sanguinarine chloride, thymol, and cinnamic acid have all been shown to inhibit different components T3SS with good efficacy [97]. In addition to directly targeting different components of T3SS, Kim et al. showed that small molecule targeting of secretion system transcription factors (LcrF) in *Yersinia* with *N*-hydroxybenzimidazoles displayed anti-virulence capabilities [98].

Lastly, several reports also reveal the successful employment of monoclonal antibodies (mAbs) targeting the needle-tip protein of the T3SS in both in vivo and in vitro model systems. For example, it has been shown that the targeting of the LcrV protein (T3SS needle cap protein) in *Yersinia* spp. functions by blocking effector protein translocation [99,100,101]. It was these findings that laid the foundation for the advancement of anti-needle tip mAb into human clinical trials [102]. One of these therapies utilizes a bi-functional anti-PcrV and -PsI mAbs for the treatment of *P. aeruginosa* infections [103,104,105,106]. The EVADE clinical trial (Clinicaltrials.gov: NCT02696902) assessing safety, efficacy, and pharmacokinetics revealed that even though patients received a 1500 mg dose of the bivalent, bispecific, monoclonal antibody (gremubamab; MEDI3902), they did not reduce *P. aeruginosa* nosocomial pneumonia incidence in mechanically ventilated subjects [107].

## 3. Targeting Quorum Sensing

Quorum sensing is a process through which bacteria change gene expression profiles according to fluctuations in cell-population density [108]. Autoinducers (AI) are the small signaling molecules that enable communication within and between bacterial species and are produced at basal levels [109]. Generally, AIs increase in a concentration-dependent manner as a function of cell density. For example, when population density reaches a critical threshold concentration, individual bacteria begin to organize into clusters that coordinate and carry out a wide range of different virulent functions, such as toxin production, antibiotic secretion, motility, biofilm formation, quiescence, sporulation, conjugation, and competence. Both GNB and Gram-positive bacteria (GPB) employ QS as a method of communication [110,111,112]. GNB uses fatty acid derivatives such as N-acylated homoserine lactones (AHL) as an AI, whereas GPB mainly utilizes processed oligopeptides [108].

**Figure 2:**
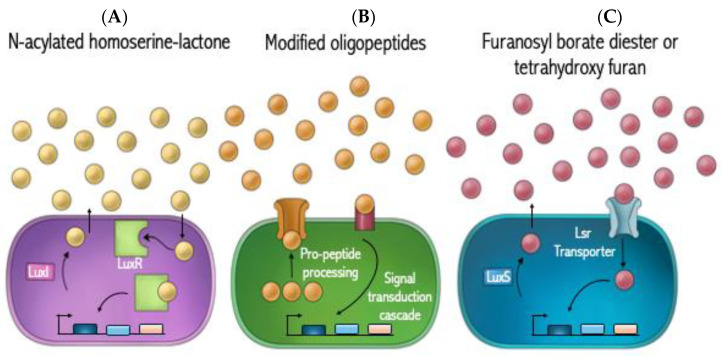
To date, three different quorum-sensing mechanisms have been identified. LuxI/LuxR in GNB and homoserine lactones as autoinducers (**A**). Autoinducing oligopeptides in GPB, which induce a signal transduction cascade that leads to altered gene expression (**B**); and lastly, the LuxS/Lsr transporter system that is employed by both GPB and GNB and the autoinducer is furanone (**C**).

Mechanistically, there exist three processes of quorum sensing: (A) LuxI/LuxR in GNB and homoserine lactones as autoinducer; (B) The AI comprises processed oligopeptides secreted by GPB; and (C) LuxS/Lsr transporter in both GPB and GNB, as well as the autoinducer, which are furanone (Figure 2). In GPB, the process of QS includes the synthesis of pro-peptidic autoinducing proteins that undergo extensive post-translational modification, such as enzymatic cleavage, the addition of lactone, thiolactone, lanthionine or isoprenyl groups, and export into the extracellular milieu. These post-translational modifications give rise to diverse structures that bind specifically to their cognate receptors. As the concentration of autoinducing peptides (AIP) increases extracellularly, the autoinducers subsequently begin to bind their specific membrane-spanning receptors and induce intracellular signaling cascades that regulate gene expression responsible for transitioning to a virulent state. In contrast to GPB, QS in GNB includes the synthesis of three core components: (A) LuxI type synthase molecule, (B) AHL signaling molecule, and (C) LuxR type receptor binding. The autoinducer for GNB is AHL, synthesized by the LuxI gene locus. Upon induction of LuxI gene expression, the mature autoinducer is synthesized and begins to passively diffuse across the bacterial plasma membrane in both directions. When enough AI molecules reach a specific threshold stimulatory concentration, LuxR gene expression is initiated to synchronously activate a panel of target genes responsible for pathogenesis. Lastly, there is a third QS mechanism that is employed by both GPB and GNB. This is known as the LuxS/Lsr transport system. The AIs are furanone that is synthesized by the same pathways that regulate LuxS synthesis. In this case, the AI defuses out of the cell and reenters through the Lsr transporter and regulates gene expression. The recent explosion in our understanding of cell-to-cell communication has established that numerous bacteria communicate by a variety of secreted signaling molecules known as autoinducers. These autoinducers coordinate group behavior to display increased pathogenic behavior [108,113,114].

The infamous GNB *P. aeruginosa* employs two AI synthase genes (*lasI* and *rhlI*), which are responsible for regulating QS and pathogenic capacity during infection [115]. In patients with cystic fibrosis (CF), *P. aeruginosa* forms complex alveolar biofilms that efficiently transport nutrients between microcolonies while simultaneously affording protection to biofilm inhabiting cells. Following the initial colonization of the lungs, individual *P. aeruginosa* cells begin to replicate and secrete two separate species-specific AIs (N-(3-oxo-dodecanoyl)-l-homoserine lactone (OdDHL) and N-butyryl-l-homoserine lactone (BHL)). As the pathogen continues to produce AI signals, extracellular concentrations begin to exceed intracellular levels, and the two AHLs reach a specific threshold concentration that enables them to bind to their specific receptors, LasR and RhlR, respectively, and initiate gene expression associated with microbial pathogenesis such as alveolar biofilm formation and production of virulence factors. Upon reaching a certain critical concentration within the biofilm, all biofilm production comes to a halt, and the second QS mechanism transitions *P. aeruginosa* through the next stage of its infectious cycle. The *rhl* QS system regulates swarming motility and production of the virulence factors rhamnolipid and pyocyanine, and it participates during the early phases of biofilm development. The *las* system regulates the gene locus that encodes various other virulence factors such as elastase, alkaline protease, endotoxin A, and different biofilm-associated genes [115,116,117]. Accordingly, these QS signaling molecules serve as “high-value” therapeutic targets for the treatment of certain bacterial infections. Several studies have shown that the inhibition of bacterial QS capabilities results in the disruption of virulent characteristics and serves as a practical target for the research and development of novel antimicrobials. There are a variety of methods used to inhibit QS pathways: (A) inhibition of autoinducer synthesis; (B) autoinducer receptor antagonism; (C) downstream inhibition of receptor binding; (D) neutralization of autoinducers using mAbs; (E) enzymatic degradation of autoinducers; (F) inhibition of autoinducer transport/secretion; and lastly, (G) mAbs that block autoinducer receptors [108,112,118,119,120,121,122,123,124,125,126,127,128,129,130,131,132,133,134,135,136]. It should be noted that not all different types of inhibitory mechanisms have been explored in the various potential pathways identified.

### 3.1. AHL Inhibitors

Several plant species (e.g., tomato, rice, pea, etc.) and soil bacteria have evolved with the ability to alter AHL activity, and the marine algae *Delisea pulchra* has been shown to secrete halogenated furanones that inhibit QS signaling. Furanones are QS signal-mimics that appear to inhibit AHL-specific regulated gene expression by binding to AHL receptor proteins in a variety of GNB [137,138], suggesting that furanones have the capability to inhibit QS-mediated biofilm formation by AHL-producing bacteria in natural environments. Ongoing efforts include the synthesis of furanone chemical analogs to prevent QS-specific propagation by *P. aeruginosa* in marine environments.

Phenolic compounds produced by edible herbs, fruits, and vegetables have been shown to possess QS inhibitory properties [139,140,141,142,143,144]. Molecules such as flavonoids are a major class of phenolic compounds and are the most diverse compounds in the plant kingdom, with over 8000 flavonoids distributed in nature. The main classes of phenolic compounds include phenolic acid, lignans, tannins, stilbene, and flavonoids. Flavonoids are the largest class of phenolic compounds. They are formed by two benzene rings linked by a heterocyclic pyran ring and are subdivided into six subclasses [139]. Some of their major contributions to plants include repelling or attracting different organisms and affording protection via their antimicrobial activities [140,145]. Recent studies reveal that numerous plant-derived compounds interfere with QS regulation by inactivating or competing for binding to receptor proteins [146]. For example, Paczokowski et al. identified that flavonoids such as phloretin, chrysin, naringenin, quercetin, baicalein, apigenin, 7,8-dihydroxyflavone, 3,5,7-trihydroxyflavone and pinocembrin all function by allosteric mechanisms that inhibit QS without binding to the autoinducer binding site [147]. Furthermore, Hernando-Amado et al. demonstrated that competition assays between cognate 3-oxo-C12-HSL and naringenin displayed inhibitory functions against the QS receptor of *P. aeruginosa* in a time-dependent manner [148]. It should be noted that the efficient use of phenolic compounds as therapeutic and functional agents and bioavailability features such as stability need to be better understood and improved.

There are three different classes of naturally occurring degradative enzymes known to target AHL signaling: lactonases and acylases. The first study of AHL-inactivating molecules was reported by Yu et al. in 2000. Yu and colleagues screened over 500 different isolates and laboratory strains and identified the *aiiA* gene (autoinducer inactivation gene) derived from *Bacillus* spp., exhibiting AHL inactivation properties. Additionally, other reports show that the *aiiA* gene is encoded by numerous *Bacillus* spp. [149,150,151,152]. The gene encoded a HXHXD sequence motif commonly associated with metallo-β-lactamases. Their study showed that purified AiiA protein inactivated multiple AHLs in vitro and functioned as a lactonase [153,154]. Lactonases are metalloproteins with the capability to hydrolyze the ester bond of the homoserine lactone ring structure to yield acyl-homoserine molecules [155,156]. Lactonases have a broad spectrum of activity and AHL substrate specificity. This is due to a highly conserved homoserine lactone ring present in all AHL molecules, of which the variable acyl chain makes nonspecific interactions with the active site of the enzyme [150,156]. Following these initial studies, different published reports reveal that heterologous expression of *aiiA* genes in various pathogens such as *P. aeruginosa*, *Vibrio cholerae,* and *Burkholderia thailandensis* decreased AHL production and altered QS-dependent behaviors [157,158,159,160].

In addition to bacterial-derived lactonases, various mammalian enzymes such as paraoxonases 1, 2, and 3 (PON1 to 3) have also exhibited AHL lactonase activity [161,162]. Each PON enzyme displays different unique activities, with PON1 and PON3 being produced by the liver and sera, whereas PON2 is an enzyme produced in various tissues. Evolutionary comparisons of PON reactivity with numerous substrates demonstrated that PON1 and PON3 act on a wider range of substrates, such as hydrolyzed lactones, esters, and phosphotriesters, while PON2 is the most active against AHLs [163,164].

Acylase enzymes also possess unique specificities for AHL molecules, which are very similar to AHL lactonases. AHL acylase activity was originally identified in the betaproteobacterium *Variovorax paradoxus*, which displayed degradative capabilities against multiple AHLs used as an energy and nitrogen source [165]. Following its initial discovery, acylase activity was also found by another betaproteobacterium, a *Ralstonia* isolate, capable of AHL inactivation as a result of *aiiD* gene expression [166]. The AiiD protein shares similarities with aculeacin (AAC) derived from *Actinoplanes utahensis,* as well as other acylases exhibiting hydrolytic capabilities against penicillin and cephalosporin [166]. It is reported that AiiD homologs are also found in several other *Ralstonia* spp. and *Pseudomonas* spp. and were confirmed to show AHL acylase activity [167,168,169,170,171,172]. Biochemical confirmation indicated that purified AiiD protein functions as an AHL acylase that releases HSL 3-oxodecanoic acid as the major degradation product [166]. AiiD expression in *P. aeruginosa* regulates pathogenesis by reducing virulence factor production and swarming motility [165,166,173]. The enzyme belongs to the oxidoreductase class of compounds that do not degrade AHL; rather, it is structurally modified by oxidation or reduction of the acyl side chain and renders it inactive [173,174]. Unfortunately, studies of different AHL acylases have not increased our understanding of the potential of these enzymes as novel therapeutic agents in vivo [155].

In addition to small molecule inhibitors and naturally occurring compounds, monoclonal antibodies (mAb) have also been employed to inhibit AHL-mediated activity. Published reports reveal moderate to high inhibitory capacity, suggesting that mAbs, as QS quenchers, have good potential for therapeutic purposes [175,176,177]. Several of the mAbs generated against the 3-oxo-hapten demonstrated good to excellent affinity (K_d_) 150 nM to 5 µM) [175].

### 3.2. AIP Inhibitors

Upon AIP binding to its cognate receptor, histidine sensor kinase protein is activated, and results in the autophosphorylation of histidine residues. Subsequently, phosphoryl groups are transferred to response regulators by phosphorylating conserved aspartate residues, which in turn activate several genes that regulate QS. Additionally, the activation of the response-regulator protein initiates the translation of signal precursors that produce the mature autoinducer signals [108,178,179]. Based on AIP-specific pathways one can envision that the inhibition of several key steps would result in the shutting down of QS regulatory mechanisms such as (A) antagonists that bind and interfere with AIP receptors, (B) kinase inhibitors after receptor binding, (C) phosphoryl transfer inhibitors of receptor histidine residues to aspartate response regulator, (D) inhibitors of post-translation modification and cleavage enzymes of AIP precursors, and (E) inhibitors of AIP efflux transporters.

Via high-throughput screening of both synthetic and natural compounds, Matsushita and Janda identified several histidine kinase inhibitors. The high-throughput screening identified two compounds known as 5 and 6, which appeared to be general two-component inhibitors of autophosphorylation activity of soluble histidine kinases. The two compounds were also found to inhibit transmembrane kinases at 50 μg/mL for compounds 5 (112 μM) and 6 (124 μM) and completely inhibited phosphorylation in *P. aeruginosa* [180,181]. Scientists at the R.W. Johnson Pharmaceutical Research Institute showed that compounds that are part of the hydrophobic tyramine inhibitor family also had inhibitory capabilities targeting two-component QS systems. These compounds inhibited the incorporation of phosphate from ATP into kinase in *B. subtilis* with IC_50_ values of approximately 1.6 μM. Kinetic analysis of one of their compounds, RWJ-49815, competed with ATP binding [182].

Library screening performed by Kjelleberg and colleagues identified two compounds from the salicylanilides family, closantel and tetrachlorosalicylanilide, as a new class of potent inhibitors of the two-component system and were shown to inhibit autophosphorylation with an IC_50_ of 3.8 μM for closantel and 45 μM for tetrachlorosalicylanilide [183,184]. The mechanism of these inhibitors causes structural alterations of the carboxyl catalytic domain of the sensor kinase that lead to the aggregation or disruption of membrane integrity [121,184,185,186]. In addition to high-throughput screening of inhibitors, the natural product open-ring Zerumbone also showed inhibitory activity against autophosphorylation of histidine kinase. The IC_50_ value for this compound is 64 µM [187]. Additionally, Kitayama et al. used a tryptophan derivative of Zerumbone and showed that it also had inhibitory effects against histidine-kinase with an IC50 value of 44 μM. This compound was shown to have inhibitory effects on methicillin-resistant *Staphylococcus aureus* (MRSA) with MIC of 100 mg/mL and IC_50_ of 200 µM and vancomycin-resistant *Enterococcus faecalis* (VRE) with MIC 50 mg/mL and IC_50_ of 100 µM. Interestingly, open-ring Zerumbone did not show efficacy against MRSA and VRE [187].

The section above is focused on histidine kinase inhibitors, whereas the following section will concentrate on AIP receptor inhibitors or agonists. Using a transthioesterification strategy, Muir et al. showed that a truncated derivative of the AIP-II thiolactone peptide had broad-spectrum antagonistic activity against *S. aureus* [188,189]. The IC_50_ values of the thiolactone peptide against four different *S. aureus* strains are 272 ± 67, 209 ± 39, 10 ± 1, and 188 ± 50 nM. Since AIP are small ligand peptides, Karathanasi et al. revealed that linear peptidomimetics have the capability to function as specific and potent antagonists of the *S. aureus* QS [190]. By using the chemical architecture of naturally occurring AIP-1 as a scaffold, Scott et al. utilized structure-activity relationship studies to synthesize several potent macrocyclic thiolactone peptide inhibitors capable of antagonizing *S. aureus* [191]. In a different study, Wright et al. identified the binding of AIPs and their receptors is driven by highly conserved hydrophobic interactions involving the C-terminal residues in AIP mediates ligand-receptor recognition for the activation or inhibition of QS [192]. Using structure-activity relationships, Vasquez and Blackwell identified a novel AIP mimetic peptide, Bnc3, with sub-nanomolar inhibitory activity against *S. aureus*. Their study revealed the presence of both hydrophobic and hydrophilic residues responsible for antagonistic characteristics, which is very similar to previously published reports about peptide-derived inhibitors [193].

A study published by Naga et al. targeting LuxR enzyme reveals that 4,5 dihydroxy-2,3- pentanedione (DPD) and L-homocysteine have the capability to disrupt AI-2 and inhibit microbial pathogenesis [120,194]. Additionally, a study published by Park et al. shows that new bicyclic brominated furanones demonstrate potent inhibitory activity of AI-2-mediated QS and subsequent biofilm formation [195]. Benneche and colleagues demonstrated that different combinations of brominated furanone have the ability to inhibit QS-associated processes, specifically a 57% reduction of biofilm development by *Staphylococcus epidermidis*. Liu et al. showed that the naturally occurring compound surfactin isolates from *B. subtilis* had the potential to inhibit AI-2 QS-driven biofilm formation by 70% [196].

There have been several reports of employing mAb as antagonists. For example, early on, Janda et al. utilized autoinducer mAb that could directly inhibit QS by the sequestration of AIP-4 elaborated by *S. aureus*. This antibody was developed by synthesizing a hapten of AP-4 conjugated to both keyhole limpet hemocyanin and to a protein carrier such as bovine serum albumin (BSA). Out of 20 mAb, one possessed high binding affinity (Kd = 90 nM) and specificity to AIP-4 while exhibiting low non-specific binding to other AIPs. Naturally accruing AIP-4 mediated competition assays showed a reversal of the anti-quorum quenching capabilities of the mAb. In vivo studies reveal that the mAb had the capabilities to protect against a lethal *S. aureus* challenge completely [134].

The efficacy of quorum quenching molecules has demonstrated good efficacy in animal models and is now being considered for coating various medical devices to prevent bacterial infections. For example, some of the first studies using anti-QS compounds were considered for the functionalization of catheters. Studies by Hume et al. reveal that covalent attachment of furanone decreased biofilm formation by *S. epidermitis* for 65 days in an in vivo sheep model [197]. Later, human studies involving 960 adult patients in intensive care units implanted with an anti-infective external coating of central venous catheters in a randomized, noninferiority trial comparing 5-fluorouracil with chlorhexidine/silver sulfadiazine in preventing catheter colonization coated central venous catheters were demonstrated to be efficient and comparable to the classically used chlorohexidine/silver sulfadiazine coated catheters [198]. Many studies have been performed showing good clinical outcomes with medical devices coated, thereby increasing our arsenal of anti-infective agents [199]. Lastly, some clinical studies reveal that anti-QS agents have increased toxicity and less stability compared to their antibiotic counterparts, therefore limiting their use [200,201].

## 4. Targeting Biofilm Formation

**Figure 3:**
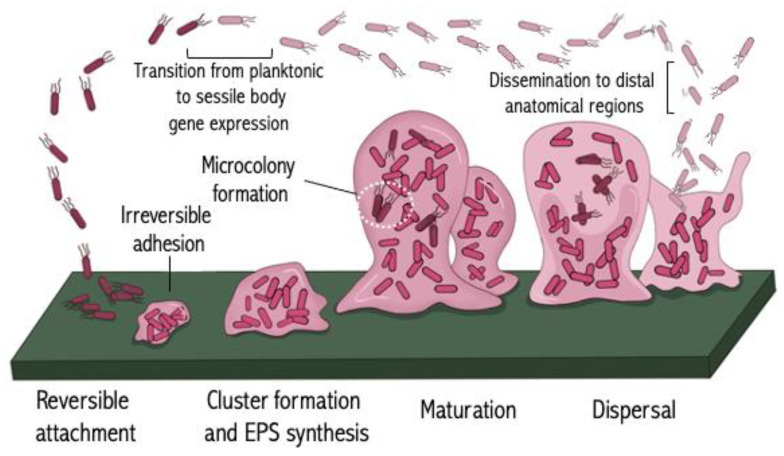
Biofilm formation is a five-step process. The first is reversible attachment, followed by irreversible adhesion. Next, the bacteria begin forming clusters and synthesizing an extracellular polymeric substance. As the biofilm matures, sessile cells begin to form microcolonies, with each microcolony assigned its own task within the biofilm community. Once the biofilm reaches a critical mass, environmental cues induce the bacteria to produce degradative enzymes to enable dispersal to distal anatomical regions.

Biofilm-producing bacteria are responsible for approximately 60–80% of chronic infectious diseases [202]. Biofilms are comprised of immobile clusters of bacteria embedded in a self-produced matrix that are tightly adherent to both biotic and abiotic surfaces. Bacteria can form biofilms on tissues such as the lungs of CF patients, as well as on medical devices and implants [53,203,204,205,206,207]. These encased microbial communities are ubiquitous in nature and can be either single species or polymicrobial, with the inclusion of *Candida* [208]. The biofilm matrix varies among bacterial species and generally comprises extracellular DNA (eDNA), lipids, polysaccharides, and protein-like substances such as alginate and fibrin [202,203]. In contrast to their planktonic state, biofilm-encased bacteria exhibit completely altered gene expression, protein production, and metabolic profiles that result in reduced rates of cell division [209,210,211]. Typically, biofilm-associated infections are persistent infections that will not get resolved by the immune response, nor will they respond to antimicrobial therapy, making treatment even more challenging [212]. In addition to serving as a protective barrier against antibiotics, biofilms also enable embedded organisms to survive host defenses such as phagocytosis [213]. During biofilm formation, both innate and adaptive immune responses are triggered, with minimal capacity to clear the infection. Instead, host tissue damage occurs. Biofilm formation is a cyclic process comprising five different developmental stages: (A) reversible attachment; (B) irreversible adhesion; (C) cluster formation and extracellular polymeric substance synthesis; (D) maturation and microcolony formation; and (E) dispersal (Figure 3) [209].

The process of biofilm formation starts when planktonic bacteria colonize a surface and utilize various forms of motility to move around, including Brownian motion, flagellar, and pili-dependent movement. In addition to flagella-mediated roaming, swimming, and swarming, bacteria also display clustering and twitching motility, which is dependent on type IV pili [214,215]. Chemotaxis, QS, and secondary signaling molecules influence bacterial movement toward a particular stimulus [109,113,216,217]. Adhesion molecules such as protein adhesins, pili, amyloid fibers, and capsules are the molecules responsible for triggering a shift in planktonic bacteria to a sessile biofilm lifestyle [218]. Initial contact with a surface is transient and reversible. Upon adhering sufficiently long to a surface, contact-dependent signals alter gene expression profiles, and the bacterium begins to transition to an irreversible adhesive state [209]. Several pathogens, including *Neisseria meningitidis*, *P. aeruginosa*, and enterotoxigenic *E. coli* (ETEC), all display contact-dependent transcriptional changes [219,220,221]. Regulation of gene expression is dictated by environmental cues such as aerobic conditions, pH, osmolality, and nutrient availability. Studies with *Salmonella* reveal that adhesion and biofilm formation are temperature, oxygen, and surface composition dependent [222]. Microcolonies are subpopulations that begin to assemble shortly after bacteria display clustering behavior and growth. The extracellular polymeric substances (EPS) are responsible for clustering and microcolony formation, whereas population growth and structural organization within the biofilm are mediated by symbiotic relationships established between different microcolonies [223]. During the biofilm maturation stage, various phenotypic changes occur to distinct microbial communities, even though the different subpopulations can be genetically identical. These changes significantly increase the ability of the bacteria to persist in a host and complicate treatment. During the maturation of a biofilm, different populations can persist for extended periods of time [224]. However, as the biofilm reaches its full maturity, the inhabiting communities can begin to disseminate from biomass to surrounding tissue. These dispersed bacteria can then seed distal anatomical regions and begin the whole process of biofilm formation or remain planktonic. For example, the formation and dispersal of dental biofilms have been linked to a variety of conditions, such as infective endocarditis and pulmonary infection [225,226].

Bacteria adapt to changing host microenvironments by employing a variety of strategies to promptly regulate biofilm formation. Pili-driven motility, quorum sensing, intracellular cyclic dinucleotide signaling pathways, membrane-bound receptors, two-component regulatory systems, and chemotactic factors all work in synchrony to regulate the space and temporal expansion of complex biofilms [227,228]. Biofilm inhibition strategies generally target either the formation stage or the mature biofilm through the disintegration of mechanisms such as biofilm matrix formation, piliation, adhesion, signaling pathways, and regulation of microbial dispersal.

### 4.1. Anti-Second Messengers

The targeting of the ubiquitous second messenger cyclic dimeric guanosine monophosphate (c-di-GMP) mediated biofilm formation is a promising approach due to its effects on critical bacterial functions without affecting growth, thereby preventing the development of drug resistance. The c-di-GMP signaling network is an incredibly complex network of signaling in bacteria, with over 100 c-di-GMP metabolizing proteins in some species. Although this regulatory circuit is absent in some bacterial species, it is present in several human pathogens, including GNB and GPB, such as *P. aeruginosa*, *Salmonella typhimurium*, *E. coli*, *V. cholerae*, *Clostridia* spp. and *Mycobacteria* spp., all of which express numerous c-di-GMP metabolizing proteins [229]. Even though c-di-GMP is involved in various biological processes, its prominent role in pathogenesis is associated with biofilm formation and suppression of motility [230,231,232]. Small molecule targeting of c-di-GMP results in the inhibition of bacterial transition from planktonic to a sessile lifestyle, which is required for biofilm formation [230].

Signaling through c-di-GMP induces the gene expression of exopolysaccharides responsible for EPS formation, pili-mediated motility, adhesion, and surface anchoring, as well as cell death and expulsion of extracellular DNA, all of which are necessary for the development of a mature three-dimensional biofilm [233]. There are ways to interfere with second messenger signaling networks, including manipulation of metabolizing activity and direct inactivation of second messengers. Reports show that intrinsic induction of specific c-di-GMP phosphodiesterases inhibits biofilm development, leading to dispersal through ectopic expression of phosphodiesterases in biofilm-forming bacteria such as *S. typhimurium*, *E. coli*, *P. aeruginosa,* and *Clostridium difficile* [230,234,235,236,237]. Since c-di-GMP is generally a diffusible molecule, data reveal that signaling is initiated by association with receptor binding, thereby blocking second messenger binding to high-affinity receptors, removing c-di-GMP from the signaling network, leading to biofilm dispersal [238,239].

Naturally occurring proteins and small molecules have also been shown to downregulate c-di-GMP levels in a cell. Studies by Kim et al. reveal that terrain, produced by *Aspergillus terreus*, is a dual inhibitor of QS and c-di-GMP signaling [240]. Using high-throughput screening methods, Anderson et al. identified hydrazonodiaminopyrazole as a potent inhibitor of c-di-GMP signaling in *P. aeruginosa*, resulting in an approximately 90% reduction in biofilm formation [241]. Xuan and colleagues designed and synthesized a series of benzothiazole and quinoline derivatives as c-di-GMP G-quadruplex inducers and identified a compound known as 5 h that exhibited biofilm inhibitory activity at 1.5 μM [242].

In addition to targeting c-di-GMP directly, other investigators sought to inhibit the enzymes responsible for its synthesis (diguanylate cyclases (DGCs)). Using high-throughput screening of the National Institutes of Health (NIH) Clinical Collection 1 (NCC1) library, Lieberman et al. identified ebselen as a favorable inhibitor [243]. After screening 1500 FDA-approved drugs in the DrugBank database through a virtual screening method to find competitive inhibitors binding to the A-site of the DGC, Wiggers, and colleagues identified the anti-inflammatory sulfasalazine and the anti-hypersensitive eprosartan as potential candidates. The IC_50_ values of sulfasalazine were in the range between 200 μM and 360 μM, and those of eprosartan were 170 μM to 888 μM. Their data showed that both compounds decreased aggregation and biofilm formation of *E. coli* in culture [244]. Fernicola et al. reported the results of a ZINC database screen where they identified two drug-like compounds with a catechol moiety and a sulfonohydrazide scaffold that competitively inhibited the PleD enzyme, part of the DGC pathway [245]. Taken together, strategies of downregulating c-d-GMP concentrations, either directly or indirectly, in a pathogen can be used as potential therapies for biofilm formation.

### 4.2. Antiadhesion

The earliest step in biofilm formation is attachment, followed by irreversible adhesion of planktonic cells to inert or biological surfaces. Accordingly, one of the first antibiofilm strategies includes the targeting of bacterial adhesion to medical devices or tissues. Antiadhesion-based therapies are attractive alternatives to antibiotics as they will not directly target critical processes that result in the development of resistance [204,246,247,248]. Both GNB and GPB produce membrane-bound proteins with adhesive properties.

Most bacterial adhesins are comprised of thin thread-like organelles known as fimbriae or pili. In addition to their adhesive properties, these appendages play important roles in conjugation, twitching motility, biofilm formation, and immunomodulation [249]. In GNB, these structures are assembled from noncovalent polymerization of various subunits with the help of Chaperone-Ushers (CU) proteins, whereas, in the case of GPB, heteromeric or dimeric pili are assembled from covalent linkages afforded by transpeptidase enzymes [250]. The significance of these adhesins in virulence is well documented and considered valuable targets for the development of biofilm inhibitors.

#### 4.2.1. Gram-Negative Bacteria Anti-Adhesins

The most widely investigated system for blocking the adhesion of GNB to host epithelial cells is various forms of pathogenic *E. coli* isolates, specifically uropathogenic *E. coli* (UPEC). Their mannose-specific appendages play critical roles in different types of infection by mediating enhanced adhesion, invasion, and colonization of epithelial tissues in the bladder and other anatomic regions [251]. Pili in GNB are classified into five different types based on their assembly mechanisms. They are generally CU pili, such as Pap pili and Fim pili, Curli, Type IV pili, and Type V pili [252]. Adhesive pili are assembled by the coordinated action of chaperone-usher pathway (CUP) proteins, which are virulence determinants. Many GNB encodes multiple CUP assembled pili, many of which are disease-associated and include Type I, P, and S pili [253]. Similar to other CUP-constructed pili, they contain a mannose-binding adhesin (FimH) at their tip that plays an essential role in host-pathogen interactions, biofilm synthesis, and the establishment of recurrent infections [254]. It should be noted that even though type I pili play a critical role in the colonization of tissues such as the murine urinary tract, their sole expression is not sufficient for long-term colonization [253,255]. Studies have shown that FimH is critical for UPEC adhesive capabilities, and its inhibition increases the susceptibility of MDR *E. coli* to antimicrobials.

Various studies have shown that compounds such as mannosides, mono- and polyvalent antagonists, anomeric phenylaglycons, glycofullerenes, trifluoperazine, thioridazine, and other small molecules display potent antiadhesive capabilities [251,256,257,258,259,260,261,262,263,264,265]. It is well established that type IV pili contribute to bacterial virulence and are necessary for pathogenesis. PilB ATPase is essential for type IV pili assembly into filamentous form. Using high-throughput screening of 2320 compounds, Dye et al. identified two PilB inhibitors in vitro, followed by an assessment of efficacy in vivo. Their data showed that benserazide and levodopa, two approved drugs for Parkinson’s disease, had strong inhibitory activity against *Myxoccocus xanthus* and *Acinetobacter nosocomialis* motility. Both compounds exhibited inhibitory activity in a dose-dependent manner [266]. Additionally, reports show the use of antibodies against FimH as potent inhibitors [261,267,268]. Furthermore, studies by Tursi et al. revealed that the use of a human mAb with pan-amyloid binding activity (mAb 3H3) against an epitope present on curli fibers had the capability of disrupting biofilms established by *Salmonella enterica* serovar Typhimurium in vitro and in vivo [269].

Prevention of piliation and motility via altered regulation and assembly with low-molecular-weight compounds such as pilicides have also been reported. Pilicides were first mentioned by Svensson et al. as potential alternative antimicrobials to conventional antibiotics [270]. Pilicides function by interfering with the CUP responsible for pili assembly [270,271,272,273,274,275,276]. For example, Cegelski and colleagues identified two ring-fused 2-pyridones with inhibitory capabilities against both type1-dependent and curli-mediated biofilm formation [277].

#### 4.2.2. Gram-Positive Bacteria Anti-Adhesins

Multidrug-resistant GPB pathogens such as *Staphylococcus epidermidis* and *S. aureus* are well known for their biofilm formation capabilities on abiotic and biotic surfaces [278]. *S. aureus* and *S. epidermidis* possess a wide range of adhesion mechanisms, enabling these pathogens to adhere and evade the host immune system [213,279]. It is established that all Staphylococcal species express microbial surface components recognizing adhesive matrix molecules (MSCRAMMs), which play important roles in host-pathogen interaction and causing disease [280,281]. MSCRAMMs comprise two tandemly arranged IgG-like folded subdomains that drive tissue adhesion. They mediate binding to ligands via a mechanism that involves significant conformational changes that enable binding to fibronectin, fibrinogen, collagen, or elastin. The most common MSCRAMMs are the cell wall anchored clumping factors (ClfA and ClfB)—serine-aspartate dipeptide repeats (Sdr-C, -D, -E) family proteins. Bone sialoprotein-binding protein (Bbp), fibronectin-binding proteins (FnbpA, FnbpB), as well as the multi-modular cell wall anchored collagen-binding adhesin (Cna) also drive the adhesion of Staphylococcci to tissues [281,282,283,284]. The cysteine transpeptidase enzyme Sortase A (SrtA) is responsible for covalently anchoring MSCRAMMs to the cell wall of GPB.

In the past several years, many efforts have been made to develop novel compounds capable of inhibiting the biofilm formation process of GPB by interfering with their adhesive capabilities. Different indole compounds belonging to different chemical classes, such as nortopsentin analogs, topsentin analogs, and imidazothiadiazole derivatives, have been shown to be potent inhibitors of *S. aureus* biofilm formation [285,286,287]. Compounds associated with the above-mentioned inhibitors showed biofilm inhibitory concentrations (BIC_50_) lower than 1 μM. Specifically, thiazole nortopsentin analogs against *S. aureus* adhesins showed BIC_50_ values in the range of 0.4 and 0.5 µM, and 1,2,4-oxadiazolem topsentin derivative exhibited BIC_50_ of 0.2 µM. Imidazothiadiazoles compounds inhibited *S. aureus* biofilm development with BIC_50_ in the range between 0.5 and 1.8 µM. Oxadiazole topsentin inhibition of the transpeptidase enzyme sortase A (SrtA) showed IC_50_ values between 2.2 and 10 µM. In the case of SrtA inhibitors, natural products, and small molecule libraries have been screened [288]. Using Förster resonance energy transfer (FRET), Jaudzems et al. screened 50,000 compounds and identified 27 derivatives, classified into seven classes. Of these 27 compounds, five were previously reported nitriles, pyridazinones, thioamides, Michael acceptors, aryl (β-amino) ethyl ketones, and two new classes were characterized by the presence of N-hydroxy/N-amino sulfonamide group and an activated halogen group, respectively [284,289]. Furthermore, published reports by Barthels et al. demonstrate that 2-sulfonylpyrimidines are also potent inhibitors of sortase A [290]. Additionally, it should be noted that several previous studies demonstrate that β-sitosterol3-O-glucopyranoside 1, berberine chloride 2, bis(indole)- alkaloid 3, isoaaptamine 4, kurarinol 5, curcumin 6, maltol-3-O-(4′-O-cis-p-cumaroyl-6′-O-(3-hydroxy-3-methylglutaroyl)-β-glucopyranoside 7, and (−)-rosmarinic acid 8 were all potent sortase inhibitors [291,292,293,294,295,296,297,298,299,300]. Lastly, studies by Wang et al. revealed that the compound eriodictyol inhibited sortase A activity and protected mice from MRSA-induced pneumonia [301].

With reference to clumping factors, initial screens performed by Prencipe et al. showed allantodapsone as a potent inhibitor of ClfA and ClfB in *S. aureus* adhesion to fibrinogen, loricrin, and cytokeratin 10 [302]. Hall et al. reported that an anti-ClfA specific monoclonal antibody (Mab 12-9) has protective capabilities against *S. aureus*. Their results reveal Mab 12-9 inhibited 90% of binding and displaced approximately 35% of already adherent *S. aureus* in murine models [303]. Additionally, a prophylactic efficacy study in rabbits by Nguyen et al. revealed that anti-Hla/Luk/ClfA mAb combination, but not those pretreated with non-specific isotype-matched control mAb (c-IgG), were protected against the lethal course of *S. aureus* induced septic shock [304]. Tkaczyk et al. developed high-affinity anti-ClfA mAb (11H10) with inhibitory activity against ClfA binding to fibrinogen, preventative capabilities against bacterial agglutination in human plasma, and promotion of opsonophagocytic bacterial killing (OPK). Prophylactic studies with 11H10 reveal reduced the severity of bacteremia in murine models [305]. A year later, Tkaczyk and colleagues identified a second anti-ClfA mAb (SAR114) from human tonsillar B cells with >100-fold increased affinity for three prominent ClfA variants and potent inhibition of bacterial agglutination by 112 diverse clinical isolates [306]. In addition to clumping factor inhibitors, Feuillie and colleagues demonstrated that a peptide derived from β-neurexin is also a very strong competitive inhibitor capable of efficiently blocking surface attachment, homophilic adhesion, and biofilm accumulation mediated by SdrC [307].

Lastly, the targeting of other adhesion proteins, such as SadP, in the zoonotic pathogen *Streptococcus suis* has also been achieved. For example, by using a phenylurea-modified galabiose-containing trisaccharide in a tetravalent dendrimeric scaffold, Ferrando et al. achieved inhibitory activity against SadP at picomolar levels [308]. With reference to can-mediated adhesion, Herman-Bausier et al. identified two inhibiting mAbs with competitive anti-adhesion capabilities that interact with Cna residues that are in direct contact with collagen [309]. A study presented by Davies and Marques revealed the identification of *cis*-2-decenoic acid with biofilm dispersal capabilities in several GNB and GPB, including *P. aeruginosa*, *Klebsiella pneumoniae*, *Proteus mirabilis*, *Streptococcus pyogenes*, *Bacillus subtilis*, and *S. aureus* [310]. Sambanthamoorthy and colleagues identified a novel benzimidazole compound in a small molecule screen, with capabilities of preventing biofilm formation by *P. aeruginosa* and *S. aureus* on a variety of surfaces in nanomolar concentrations [311].

According to the NIH, biofilms account for up to 80% of microbial infections. Although significant efforts have been made to maintain sterility, prosthetics, and implantable medical devices continue to become contaminated by bacteria. Medical devices such as catheters, ventilators, prostheses, and topical treatments play a significant role in the acquisition of nosocomial infections, with biofilm-forming bacteria as major contributors responsible for these severe medical complications [312]. Considering that antibiofilm compounds prevent adhesion and biofilm formation, novel medical devices coated with antibiofilm dressings are being developed to inhibit the formation of biofilms and minimize virulence. Similar to dressing medical devices with quorum quenching molecules, biofilm inhibitory compounds are also being incorporated into biomaterials in such a way that they retain antimicrobial activity upon device implantation [313].

## 5. Targeting Secreted Toxins

Both GPB and GNB produce exotoxins to enhance their survival [314]. These secreted toxins play a direct role in the pathophysiology of disease, and in some cases, such as in *V. cholerae*, they play an essential role as disease-causing entities [315,316]. Exotoxins function by a variety of mechanisms, such as directly damaging host cell membranes, mimicking host-derived proteins, binding cell surface receptors to initiate signaling cascades, and translocating into host cytoplasm to commandeer cell functions [61,316]. In all instances, the exotoxin must first establish contact with the host to exert its toxigenic effects. Inhibition of exotoxin activity has been utilized successfully as an immunization strategy and requires an in-depth understanding of key steps associated with the toxins’ mechanism of action when interacting with host cells [317,318]. This indirect approach is not bactericidal; rather, it inhibits the pathogenic capabilities of the bacteria and promotes effective clearance by the immune system without resulting in the development of drug resistance. In some cases, anti-virulence modalities make the application of conjunctive antibiotic or antibody therapy a more viable option [319,320]. In the following section, I will highlight toxin-specific, passive, and active immunization strategies employed to combat a wide array of bacterial diseases.

*V. cholerae* is a toxigenic, facultative Gram-negative anaerobe that infects the intestines of malnourished individuals. The bacterium horizontally acquired a pathogenicity island encoding two toxins known as Zonula ocludens toxin (Zot) and cholerae toxin (CT) [61]. Zot is an outer membrane-associated protein that induces a signal transduction cascade and is responsible for the disassembly of tight junctions (TJ) between epithelial cells. The second toxin elaborated by *V. cholerae* is responsible for activating adenylate cyclase—cyclic adenosine monophosphate (cAMP), which results in the alteration of sodium chloride transport and subsequent expulsion of water from the cell [61,62].

**Figure 4:**
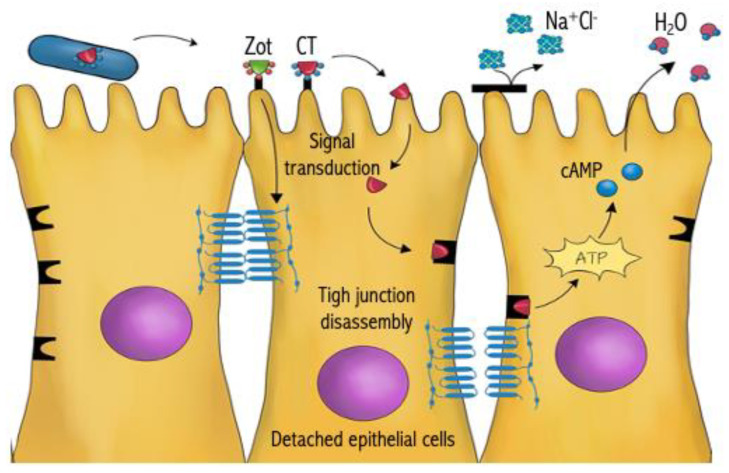
Zot and CT function in synergy when interacting with host epithelial tissue. Zot initiates a signal transduction cascade that leads to the disassembly of tight junctional complexes between epithelial tissue, resulting in a detached morphology required for CT to induce the maximal expulsion of electrolytes and water from enteric epithelial cells.

In the context of host-pathogen interaction, the synergistic effect between Zot and CT is displayed by the necessity of a detached epithelial-cell rounding morphology (induced by Zot) for maximal expulsion of electrolytes mediated by CT and the subsequent onset of acute dehydrating diarrhea commonly seen with *V. cholerae* infections (Figure 4) [315,316]. Using *V. cholerae* as an example, two strategies have been developed to minimize its virulence. The first strategy is to establish long-term immunological memory by converting the two toxins into toxoids. Toxoids are produced by heat that deactivates the toxin and treats it with a formaldehyde solution. Published reports reveal that in vivo immunization of toxoids generated a significant increase in the levels of protective, antitoxin serum antibodies [318,321,322,323,324].

**Figure 5:**
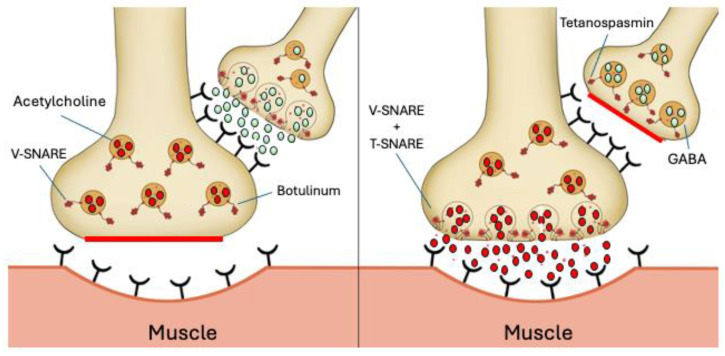
Tetanospasmin inhibits GABA, resulting in the release of acetylcholine. Tetanus toxin interferes with the fusion of GABA-carrying vesicles with the presynaptic cleft; therefore, the excitatory neurotransmitter acetylcholine is released. In contrast, botulin toxin interferes with the release of vesicles carrying the excitatory neurotransmitter acetylcholine.

*Clostridium tetani* is an anaerobic, gram-positive, spore-forming bacillus that produces two exotoxins, tetanolysin and tetanospasmin [323]. Tetanolysin is an oxygen-sensitive hemolysin that is related to streptolysin and the θ-toxin of *Clostridium perfringens* [325]. It is believed to play a part in the initial establishment of infection at the site of inoculation and does not have any other role in pathogenesis. Tetanospasmin, referred to as tetanus toxin, is considered one of the most potent neurotoxins on a weight basis [318]. Tetanus toxin is a zinc protease that inhibits synaptic vesicle fusion with the plasma membrane at the end of axons. It works by interfering with presynaptic neurotransmitter release [326]. The toxin initially enters through peripheral neurons, followed by retrograde transport into the central nervous system, where it inhibits gamma-aminobutyric acid (GABA) release (Figure 5) [327]. The toxin binds to a receptor, is internalized by endocytosis, and is transported to nerve cell bodies, primarily motor neurons in the central nervous system [328]. The mode of action of tetanus toxin is like that of another well-known toxin, botulinum toxin, which is produced by an anaerobic organism, *Clostridium botulinum* [329]. In addition to taking a prophylactic approach, such as using toxoid vaccines, neutralizing monoclonal antibodies (mAbs) is currently being developed as a therapeutic strategy used to treat the severity of illness after it is established and to prevent death from occurring [330,331,332,333]. In vivo, studies showed that the combination of 200 ng/kg of human mAb 1 (TT104) and 200 ng/kg of human mAb 2 (TT110) provided full protection of mice for 15 days, as was observed with tetanus immunoglobulin treatment [331]. A study conducted by Aliprandini et al. showed that when two mAbs targeting the heavy chain and the uncleaved whole toxin were combined, mice showed no symptoms of toxin-related toxicity when the highest amount of mAb was used (1.25 μg of each) [332].

*S. aureus* is a prevalent human pathogen affecting individuals worldwide. Its extensive antibiotic resistance profile reinforces the need for the development of alternative interventions like preventative vaccines and monoclonal antibodies targeting the numerous virulence factors elaborated by this pathogen. Following the success of currently marketed vaccines targeting different bacterial pathogens such as *Streptococcus pneumoniae*, *Haemophilus influenzae B,* and *N. meningitidis*, which use glycoconjugates to elicit a potent and long-lived immune response against their polysaccharide antigens, as opposed to classical chemical conjugation, glyco-conjugation is also being explored for preventing *S. aureus* infections. In contrast to the failures of prior *S. aureus* vaccines developed by chemical conjugation to combine a polysaccharide to carriers with unrelated proteins, glycol-conjugation to native proteins has the potential to represent a novel method of improving vaccine efficacy [334,335]. For example, experimental vaccines in which the two most common capsular serotypes in the clinic, CP5 and CP8, are glyco-conjugated to the *S. aureus* α-toxin (Hla) have shown to be promising using in vivo model systems [336,337,338]. Additionally, studies of intranasal and intramuscular administration of a combination of recombinant staphylococcal enterotoxin B (rSEB) and manganese transport protein C (rMntC), in conjunction with an adjuvant, as vaccine candidates revealed strong systemic and mucosal protective immune responses in both sepsis and pneumonia murine models [339]. To that end, it should be noted that the complexity of *S. aureus*-mediated infections, the range of secreted toxins, and the target populations affected by this pathogen requires a multipronged approach that uses a combination of different intervention strategies [340].

Reverse vaccinology is a novel approach that employs predictive modeling techniques to identify new pathogen targets that are ideally conserved among many different clinical isolates and those that display protective immune responses. To date, several vaccine development methods have been employed to target *P. aeruginosa* infections, including whole-cell vaccines [341], killed attenuated live vaccines [342,343], outer membrane vesicles [344], outer membrane complexes [345], flagella [346], pilins [347], and glycol-conjugate vaccines [348]. Despite extensive research efforts, many of these strategies have been tested experimentally in animal models. However, few have reached the clinical setting, and no FDA-approved vaccine candidate has made it to market [349,350].

Current vaccine strategies targeting *P. aeruginosa* concentrate on virulence factors since there is minimal risk of the pathogen reverting back to its virulent state, as is the case with live-attenuated or killed but metabolically active whole-cell vaccines [347]. A multi-epitope subunit vaccine comprising a series of inherent epitopes is an ideal method for the prevention and treatment of bacterial or viral infections [351]. The advantage of subunit vaccines is that they minimize impurities, increase stability, simplify tissue targeting, have feasible manufacturing scalability, and are suitable for people with compromised immunity [352]. Accordingly, reverse vaccinology enables researchers to employ genomic or proteomic analysis to identify virulence factors such as exotoxins carrying a series of immunogenic epitopes and investigate them from different aspects of antigenicity and sequence identity across a variety of pathogenic clinical isolates. One such example includes the phylogenetic tracing of the evolutionarily conserved toxin Zot, elaborated by many GNBs, including *P. aeruginosa* [316]. For example, a recent study identified a multi-epitope-based subunit vaccine candidate against Zot, secreted by *P. aeruginosa,* using different immunoinformatic and structural modeling approaches. The value of this approach is that it identified a multi-epitope subunit vaccine capable of targeting a highly conserved stretch of amino acids within the Zot protein present in all clinically relevant strains tested. Furthermore, several other studies using in silico modeling have also revealed promising vaccine candidates to prevent *P. aeruginosa* infections [353,354,355,356,357]. Taken together, reverse vaccinology platforms for vaccine design and development can be used not only against *P*. *aeruginosa* but also against other superbugs. As a result, new-generation multi-epitope vaccines can be developed based on antigens that were not previously detected or even gone unnoticed.

## 6. Bacteriophage Therapy

Phages are viruses that only infect, replicate, and destroy bacteria without harming eukaryotes such as human or animal cells. Phages are some of the smallest nucleic acid entities surrounded by a protein coat. They are omnipresent in the environment and are the most ubiquitous in the biosphere. They are very diverse morphologically and genomically. However, just like any other viral particle, all phages consist of a nucleic acid genome (DNA or RNA) encapsulated by phage-encoded capsid proteins that protect the virion until it reaches a host [358]. Phage genomes are incredibly diverse and range from 2435 to over 500,000 base pairs [359,360,361]. Phages tend to be very host-specific and usually infect a single species of bacteria, or in some instances, they can only infect specific strains [362]. Upon attachment to a susceptible host, a phage’s sole purpose is to pursue one of two replication strategies: entering lytic or lysogenic cycles [363]. During the lytic phase, the phage attaches to its host, injects its genome into the cytoplasm, and utilizes host resources to rapidly replicate autonomously and synthesize viral-associated capsid proteins. During the maturation stage, new virions are assembled, followed by the bursting of host cells to infect another susceptible host. Compared to lytic replication, the viral genome of a lysogenic phage integrates into its host genome and ensures its replication as the host genome replicates without causing any damage to the host [363].

Prior to the discovery of antibiotics, phage-specific treatment modalities were becoming very common in mediating bacterial infections in many Eastern European countries [364,365]. In contrast to the United States and most of Western Europe, the former Soviet Union and Eastern European nations vigorously pursued phage-based applications for clinical practice. The early results of such studies were primarily written in Russian, Georgian, and Polish languages and were not available to Western scientific communities [366].

Phages were first discovered in the early 1900s by two bacteriologists: Frederick William Twort and Felix d’herelle [367]. However, the first reported use of phage therapy as an antimicrobial was in 1921 by Richard Bruynoghe and Joseph Maisin, who were trying to treat staphylococcal skin disease [368]. Due to their promising results, countries such as the United States began adopting phage therapy as a common practice for treating bacterial infections. In the 1940s, companies like L’Oreal and Eli Lilly began producing several phage products for human use, including formulations to treat *E. coli* and some staphylococcal or streptococcal infections [365]. These phage therapies were being applied to numerous local infections such as abscesses, wounds, vaginitis, respiratory tract, and mastitis. However, early results were controversial, and by then, the advent of antibiotics had resulted in the halting of commercial manufacturing of phages in Western nations. Contrary to the West, many institutions in the former Soviet Union, such as the Eliava Institute of Bacteriophage, Microbiology, and Virology (EIBMV) of the Georgian Academy of Sciences, Tbilisi, Georgia, and the Hirszfeld Institute of Immunology and Experimental Therapy (HIIET) of the Polish Academy of Sciences actively continued to pursue therapeutic phage research and commercial production [369,370,371].

There are four general strategies for developing phage-based therapeutics: (A) cocktails of multiple phage components, (B) cocktails of periodically modified phages with an added spectrum of activity against new strains of target pathogen, (C) personalized phage therapy with precision against a specific MDR bacteria, and (D) genetically engineered phage therapeutics [372,373,374,375,376]. Fixed cocktail strategies are comprised of developing a set combination of phages to address the diversity within a single bacterial pathogen. This approach utilizes phage preparations as an off-the-shelf combination developed for the treatment or prevention of bacterial disease. The additive cocktail approach includes a periodic addition or replacement of phages to broaden the spectrum of host range within the complex mixtures of phages to target multiple emerging pathogens [377]. The personalized approach includes the identification of different bacteriophages with lytic activity toward a bacterial pathogen isolated from the patient [372]. The environment is an ideal source of diverse phages. However, their complex interactions with bacteria and safety concerns have rendered only a small fraction of naturally occurring phages deemed for therapeutic purposes. Additionally, the vast diversity of phages makes their detailed characterization impossible. Moreover, no single phage possesses all the necessary features to be an ideal therapeutic agent. An alternative to employing naturally occurring phages for therapeutic purposes is the targeted genome engineering of phage isolates with desired characteristics [378].

Growing MDR to conventional antibiotics has challenged the clinical employment of even the most efficacious antibiotics. The bacterial pathogens posing the greatest threat to the efficacy of antibiotics today include *Enterococcus faecium*, *S. aureus*, *Klebsiella pneumoniae*, *A. baumannii*, *P. aeruginosa*, and *Enterobacter faecium*, collectively known as the ESKAPE pathogens [379]. These ESKAPE bacteria are among the most deadly and costly pathogens responsible for the growing prevalence of MDR. With the proliferating threat of MDR, phage therapy has made a comeback to the forefront as a potential treatment modality to mitigate a rapidly increasing worldwide health crisis. The majority of phages listed in NCBI were discovered through the SEA-Phages program established by the Howard Hughes Medical Institute. This program has a comprehensive guide for discovering and characterizing phages across numerous host bacteria and sample types. Traditionally, phage discovery involves four steps: isolation, purification, amplification, and characterization. Environmental sources of phage discovery include water samples from rivers, lakes, hospital waste, and sewage treatment plants [380,381].

**Figure 6:**
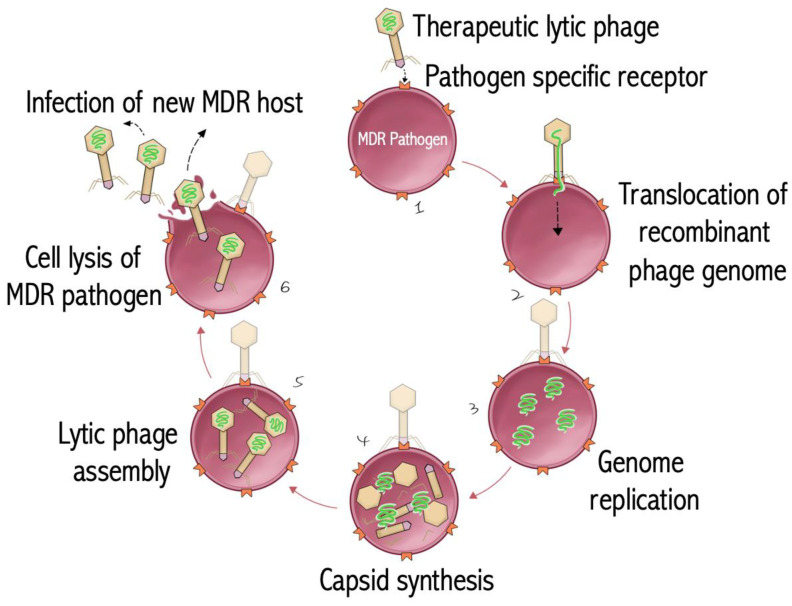
Therapeutic lytic phages undergo a simple life cycle when targeting an MDR host. As soon as the therapeutic phage attaches to its host cell-specific receptor, the lytic phage injects its genome into the cytoplasm and immediately begins commandeering host functions to synthesize as many phage virions as possible. Followed by the expression of suicide genes responsible for cell lysis and release of newly synthesized virions to infect neighboring host cells.

The development of phages as a treatment modality involves the engineered targeting of species or strain-specific pathogenetic bacteria. In this context, lytic phage genomes can be manipulated to carry bactericidal suicide genes that induce the MDR pathogen to kill itself (Figure 6). Phages are clinically significant as bactericidal agents for several reasons. First, they have been used in clinical practice for over 100 years with positive clinical outcomes. Phages are often produced as a powder formulation and can be stored without refrigeration. They can be administered to patients with intolerance to antibiotic therapies. Their use avoids dysbiosis of the microbiome by using selective targeting of MDR bacterial pathogens. Phages can be used in combination with antibiotics [382]. Generally, a single dose is required since lytic phages have a replicative capacity to increase their numbers and kill neighboring pathogens while minimizing damage to host microflora. Lastly, they are well tolerated with minimal side effects [377,381,382,383,384,385,386,387].

To date, numerous clinical trials have been initiated to evaluate the safety and efficacy of phage therapy, and these clinical studies are continuing to grow through the years. According to clinicaltrials.gov, a total of seven phage therapy trials have been conducted between 2000 and 2015. In contrast, this number has increased to 18 clinical trials initiated in 2022 alone. As of March 2023, a total of 45 clinical trials are listed on clinicaltrials.gov, and several additional phase I/II completed trials from 2015 have been listed on the European analog website. The site of application detailed in these clinical trials varies from local to systemic infections [381,388]. Several phage therapy candidates have made it to phase III trials and will most likely make it to market in approximately five years [389].

In 2005, a study conducted by The Center of Phage Therapy Unit (PTU) at the Ludwik Hirszfeld Institute of Immunology and Experimental Therapy in Poland evaluated both the efficacy and safety of phage therapy in 153 patients with chronic MDR infections and noticed a significant percentage of patients were able to tolerate therapy with good clinical outcomes [390]. Similarly, a retrospective study evaluating patients treated with phage therapy between January 2008 and December 2010 revealed that 153 patients experiencing a wide array of chronic bacterial infections unresponsive to conventional antibiotics yielded promising results [390]. Additionally, the Antibacterial Resistance Leadership Group (ARLG) reviewed various cases of phage therapy applied to 63 individuals with a life-threatening illness in single-patient trials and found that 51 cases resulted in favorable outcomes [391]. The ARLG concluded that certain natural phage formulations are well-tolerated and that they are recognized as being safe by the FDA [391]. A number of other single-patient phage therapy trials, used as an adjunct, also demonstrated promising results in targeting hard-to-treat MDR infections [392].

The first successful clinical use of intravenous phage therapy in the U.S. occurred in March 2016 at the University of California San Diego, where a phage preparation was employed to treat MDR *A. baumannii* [393,394]. Aslam et al. reported the use of intravenous application of phage therapy in ten patients inflicted with MDR infections, including *A. baumannii*, *P. aeruginosa*, *S. aureus*, and *E. coli*, and showed that seven of ten individuals experienced successful outcomes with no safety concerns [393]. A second recent study conducted by Little et al. revealed successful outcomes with phage therapy against a cutaneous *Mycobacterium chelonae* infection [395]. It was reported that the patient experienced a significant improvement in just two weeks of therapy. Given the rapid rise and significant impact of MDR bacterial pathogens, these recently successful clinical applications of phage therapy have initiated the emergency use authorization by the U.S. Food and Drug Administration (FDA) to allow treatment for the compassionate use of this modality on a case-by-case basis [381]. Lastly, phage therapy is also being investigated for the treatment of secondary bacterial infections, such as patients infected with COVID-19 and MDR infections associated with transplants [396,397,398].

To that end, there are numerous advantages associated with phage therapy. For example, bacteria that are susceptible to lytic phages are more prone to death, as compared to conventional bacteriostatic antibiotics, which may drive bacterial evolution towards resistance [399]. Phages possess auto-dosing capabilities, whereby during lytic growth, the phages increase in numbers, specifically where host density is high [400]. Since phages are ubiquitous in the environment and are comprised of mostly nucleic acids, they are inherently nontoxic [358]. However, phages can interact with the immune system, leading to rapid clearance, and highly purified phage preparations are required to avoid anaphylactic response due to endotoxin contamination [401,402,403,404]. Due to their specific host range, phages have the potential to minimally impact a patient’s microflora [401,405,406]. In contrast, the broad spectrum of conventional antibiotics can lead to the development of superbugs such as *C. difficile* colitis [407]. The narrow host range of phages minimizes the number of different bacterial types from acquiring phage-mediated resistance mechanisms [106]. This is in contrast to the significant number of bacteria that can develop resistance to general antibiotics [407]. Since phages utilize a different mechanism of action compared to antibiotics, specific antibiotic resistance mechanisms do not translate to phage resistance. As a result, phages can be employed to treat MDR *S. aureus* [401,402,406,407,408]. Lastly, phages are incredibly versatile in terms of formulation. They can be used in conjunction with antibiotics, and their application can come in various forms [409]. For example, different formulations such as liquids, lyophilized, aerosolized, encapsulated, creams, and impregnation into solids are all possibilities [410,411,412].

Not all phages are ideal for therapeutic purposes. In stark contrast to the benefits of phage therapy, this treatment modality suffers from several biological limitations. Phages are very complex since they are comprised of both protein and nucleic acid; therefore, it is difficult to evaluate their quality and curative effect. Lysogeny is another factor that also affects efficacy. In many cases, lysogenic phages can inhibit the effect of other phages on their host bacteria after integration into a host chromosome. Lysogenic phages can also horizontally transmit toxins and antibiotic resistance genes to target hosts. Moreover, there are potential risks associated with the emergence of bacterial resistance to phage therapy that can prevent viral infections. For example, there could be a loss of receptor expression, receptor conformational changes, receptor concealment, secretion of factors that prevent phage adhesion to a pathogen, and activation of measures that block phage DNA injection and replication in a host [413,414,415,416,417]. Several studies have demonstrated that numerous pathogens can undergo receptor loss or membrane protein modifications that prevent phages from carrying out their therapeutic effects [414,418,419,420]. Restriction modification systems, abortive infection, superinfection exclusion, and CRISPR/Cas are all bacterially inherent mechanisms that prevent the promise of phage therapy.

The outcome of clinical trials with different phage-host combinations is heterogeneous. Large variations exist among trial participants, and there is complete clearance of pathogens vs. none at all [390,402,421]. There exists a lack of standardization. Accordingly, clinical results of phage therapy show large variations in efficacy, making it difficult to predict the outcome of individual cases. The pharmacokinetics and pharmacodynamics of phage therapy are more complex than those of fixed composition of small molecule antibiotics, and the use of cocktails makes these issues even more complicated. Co-infections can also lead to superinfection exclusion or lysis inhibition, as well as cross-resistance. There are no comparative human studies of phage titers in plasma following administration. Ideal human dosing, metabolism, and routes of administration are yet undefined due to the potential of immune clearance [422]. Furthermore, the varying and unknown concentration of pathogens at the site of infection is also an issue. Other drawbacks include the heterogeneity of bacterial populations, inter-individual differences, and inter-phage variables such as persistence and replication, which must also be considered. There are local differences in bacterial growth and the replication of their corresponding phages, availability of nutrients, biofilm formation, and released debris, which are also impeding factors. Unlike conventional antimicrobials, which are eliminated by phase I and phase II enzymes, phages are eliminated by the reticuloendothelial system in the spleen, thereby resulting in unknown bioavailability, clearance rate, charges in hydrophobicity, binding affinity to plasma proteins such as human serum albumin, lipoproteins, and glycoproteins [423]. Taken together, pharmacological parameters of in vivo phage therapy, including phage titers, phage adsorption rate, phage decay or elimination, and diffusion rates, are all issues that must be addressed.

In addition to the inherent biological drawbacks associated with phage therapy, there are several regulatory hurdles that must also be overcome. The success of phage therapy is highly dependent on the safety of manufacturing practices. Broad medical applications require that all large-scale phage preparations be produced according to Good Manufacturing Practices (GMP) approved by regulatory bodies [424]. Production of all phage products must comply with the same stringent regulations applied to conventional pharmaceuticals to ensure high-quality standards and minimal variability between batches; however, no clear guidelines have yet been developed for phage manufacturing. To date, a clear framework that specifically defines phages as medical products for human use does not exist [425]. Several production methods have been developed to manufacture and purify phage preparation, but none have reached optimal results. Another aspect includes proper quality control. Phage stock preparations must be regularly assessed for sterility, stability, and cytotoxicity. Phages must be highly purified and clinically safe. Accordingly, the main problem in phage preparations is their separation from the remains of bacterial cells, endotoxins, exotoxins, and non-cellular compounds. Stability is a key requirement to ensure that stored formulations have consistent activity without a drop in phage titer. Additional studies are required to understand the protection given by different forms of formulations and their effects on immune clearance [426]. The high specificity of phages requires the screening of large collections of phages. High-throughput screening methods need to be developed to identify phages with desired characteristics. Moreover, robust techniques must be developed to characterize phage morphology, adsorption rate, latency, burst size, and thermal or pH stability [427]. Policies and regulations for phage therapy have not been established. Regulatory bodies have not yet created strict standards for the isolation and purification of phages, which makes the efficacy of phage preparations variable. Additionally, there is a lack of standardized procedures in clinical treatment with phages. Lastly, an important consideration regarding the clinical application of phage therapy pertains to whether the development will occur on an industrial scale or on a hospital-based, patient-specific scale.

## 7. Fecal Microflora Transplants

Our appreciation of how our body’s commensal microflora plays a role in health and disease can be traced back to the early 1900s [428]. Depending on the anatomical region, the microflora can be classified into gut, oral, respiratory, and skin. In general, these microbial communities live in a symbiotic relationship with a healthy host. A balanced microflora functions by inhibiting the colonization of pathogens, breaking down toxins, stimulating cellular differentiation, synthesizing essential vitamins, and altering the gut-brain axis [429]. Not only does the microflora protect against pathogens that breach the body’s barriers, but it also shapes the development of the immune system [430]. In recent years, many have referred to microflora as a virtual organ in the body. Each person possesses a distinct microfloral signature specific only to them, with approximately 500 to 1000 different bacterial species acquired during early life. Each species produces thousands of metabolites that influence host functions and consequently affect an individual’s fitness. When in a dysbiotic state, bodily functions become dysregulated and lead to the development of chronic health conditions such as diabetes, obesity, metabolic syndrome, neurodegeneration, and respiratory disease. The gut microflora is the most medically investigated and is considered the most significant one in maintaining our health [431]. It has been calculated that the human gastrointestinal tract is composed of 10 times more bacteria than there are human cells in the entire body. All of these factors work in synchrony to prevent disease [429,432,433].

*C. difficile* is an anaerobic, spore-forming GPB that causes infections of the colon and can lead to symptoms ranging from diarrhea to life-threatening damage to the gastrointestinal (GI) tract [434]. Clinical symptoms range from asymptomatic colonization, followed by mild diarrhea and progression to a severely debilitating disease with symptoms ranging from high fever, abdominal pain, paralytic ileus, colonic dilation, sepsis, and pseudomembranous colitis. Illness from *C. difficile* is generally associated with chronic administration of antibiotics, mostly affecting older adults and immunocompromised patients residing in hospitals or long-term care facilities. *C. difficile* is known as a nosocomial infection, but it has been shown that infection can also occur in community settings [435,436]. Community-acquired strains of the bacterium usually cause disease in younger individuals and lead to lower mortality rates. However, they are on the rise. It is estimated that case-fatality rates of *C. difficile* are approximately 6%, and of these, 35% of patients experience recurrent *C. difficile* infection [437]. Current treatment guidelines depend on whether the episode is an initial or recurring infection. The severity of the infection is also considered, depending on white blood cell counts, serum creatinine levels, and other associated signs and symptoms. Initial episodes are treated with standard antibiotic therapy, such as oral vancomycin or fidaxomicin [438,439]. However, in the case of MDR *C*. *difficile* infections and recurrent disease, fecal microfloral transplants (FMT) are recommended [440].

**Figure 7:**
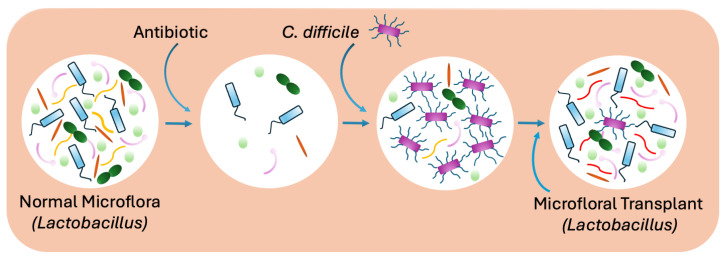
Microfloral fecal transplants reestablish symbiosis after chronic administration of antibiotics. Following heavy antibiotic treatment, the gut microflora is eradicated, enabling MDR *C. difficile* to outcompete the remaining flora and cause infection.

Once the commensal microflora of the GI tract is eradicated with the use of conventional antibiotics, *C. difficile* takes advantage of the opportunity to dominate the GI landscape due to the absence of a repressive force provided by commensal microbial inhabitants. FMT reestablishes microbial equilibrium in the patient’s GI tract by allowing the transfer of a healthy microflora that is able to suppress *C. difficile* outgrowth (Figure 7) [440,441]. The therapy generally works by replacing the protecting microflora that was disrupted by antibiotics or other environmental factors. FMT procedures include the collection of stool samples from a healthy donor and its introduction into an infected patient’s GI tract. Transplantation can be implemented to target different anatomical regions of the GI tract, such as the lower proximal, lower distal, or upper GI tract. Published reports from multiple centers reveal consistent cure rates of approximately >90% [442].

Probiotics are a century-old concept that uses live bacteria from the *Lactobacillus* spp., *Bifidobacterium* spp., and *Streptococcus* spp., as well as some yeasts, including *Saccharomyces boulardii*, that have beneficial effects on health when applied to the body [326,443,444]. It should be noted that probiotics can also include organisms that are not natural inhabitants of the human intestines, like *Lactobacillus bulgaricus* and *Streptococcus thermophilus,* which are commonly employed as starters in dairy products [445]. These microorganisms enhance gut microbial balance by promoting proper food digestion, restoring bile acids, outcompeting disease-causing pathogens, producing vitamins, or influencing an individual’s immune system. To date, several studies have suggested that probiotics can prevent and treat *C. difficile*-associated diarrhea (CDAD) in adults and children [446]. These preliminary clinical investigations reveal that the supplemental benefits of probiotics are associated with regulating the intestinal microflora by inhibiting *C. difficile* proliferation. Furthermore, published reports reveal that probiotics have the potential to reduce the incidence of *C. difficile* infections by as much as 50% in high-risk populations [326]. These studies also show that probiotics are well tolerated by all study participants with no major adverse effects. Therefore, these investigations warrant the use of probiotics as an adjuvant therapy in patients at risk for developing CDAD to optimize prophylactic use and treatment of the disease. However, these prior studies were limited by the heterogeneity of probiotic products, dosage, and patient populations. To that end, as probiotic use remains controversial, additional randomized clinical trials comparing larger sample sizes and diverse patient populations are needed to assess the benefits of optimal dosing and duration of probiotic administration during the course of intestinal infections [447].

In addition to *C. difficile* infections, FMT is also being investigated for other health conditions. For example, a study conducted by Wu et al. revealed that FMT applied to patients inflicted with both type 1 and type 2 diabetes showed improved metabolic profiles and alleviated symptoms [448,449,450,451]. With respect to food allergies, ongoing clinical trials are investigating the safety and efficacy of oral encapsulated FMT to ameliorate this allergic condition [452] (clinicaltrials.gov NCT02960074 and NCT05695261). A clinical trial conducted by Mocanu et al., evaluating the effects of oral FMT, in conjunction with low-fermentable fiber supplementation, exhibited improved insulin sensitivity in patients with severe obesity and metabolic syndrome [453]. By performing a meta-analysis of randomized clinical trials pertaining to metabolic syndrome, Qui, and colleagues report that patients experienced improvement in HbA1c, insulin sensitivity, and HDL cholesterol [454]. Furthermore, Zhang et al. reported results of a systematic review with meta-analysis based on randomized clinical trials and has also shown that FMT is a viable option for patients with metabolic syndrome [455]. However, further investigation must be conducted prior to wide use of this technique for conditions other than *C. difficile* infections.

## 8. Future Perspectives

The alarming rise of multidrug resistance (MDR) amongst numerous bacterial pathogens has outpaced the development of novel antibiotics and, as a result, has led to continued efforts to identify cutting-edge, antibiotic-independent treatment modalities. In contrast to conventional antibiotics, these new alternatives will not kill bacteria directly or inhibit their growth; rather, they disarm the pathogen and inhibit the progression of disease. This strategy is focused on utilizing compounds and techniques that inhibit virulence instead of inhibiting bacterial viability, enabling clearance of the pathogen by the host immune response or in conjunction with low-dose antibiotics. The issue with current antibiotics is that they target viability and cause selective pressures that lead to the development of MDR [456]. Accordingly, there are several advantages to targeting bacterial virulence: (A) a significant increase in the repertoire of drug targets; (B) the development of antimicrobials that utilize alternative modes of action; (C) minimizing selective pressure and the emergence of resistance; (D) enabling personalized therapies and lastly (E) preserving host microflora.

The Gram-negative bacteria (GNB) type III secretion system (T3SS) is essential for pathogenesis and is an attractive drug target. Studies have shown that any defect in assembly or dysfunction in T3SS renders the pathogen non-virulent. The needle tip of the nanoinjector apparatus functions by delivering effector proteins from the bacterial cytoplasm into host cells [457]. These T3SS share a high degree of homology with eight critical core components of flagellar T3SS and contain up to 30 proteins associated with gene expression, protein secretion, and translocation of effector proteins into host cells [458]. T3SS consists of numerous different structurally diverse proteins, each one serving as a potential drug target. To date, several T3SS small molecule inhibitors have been identified that interfere with transcriptional regulation, chaperone-effector interaction, structural assembly, and effector protein translocation without showing toxicity to eukaryotic cells [67,70,87,90,95,456,458,459,460]. It has been shown that these inhibitors block the bacteria from injecting effector proteins into host cells. However, the exact mechanism of action has not been identified, thereby delaying their advancement into the clinic. Other T3SS inhibitors include vaccines, polymers, monoclonal antibodies, and polypeptide mimics [458]. The development of monoclonal and bispecific antibodies targeting *P. aeruginosa* T3SS tip protein showed promising early potential in the treatment of patients with lung-associated infection but failed to meet efficacy endpoints in phase II clinical trials [104,105,461,462,463]. Although early clinical studies did not show the desired outcomes, the clinical application of T3SS inhibitors remains to be further exploited. It should be stressed that T3SS inhibitors will not stop bacterial proliferation. Therefore, bacteria must be cleared by other means, such as the host immune response or conjunctive low-dose antibiotic treatments, which may lead to a clearance of pathogens and increased immune cell memory.

Bacterial quorum sensing (QS) is a form of cell-to-cell communication in which specific genes are activated to coordinate pathogenicity and acclimation to a continuously changing host environment. There are three families of QS signals consisting of acyl-homoserine lactone (AHL), autoinducing peptide (AIP), and autoinducer-2 (AI-2) [464]. These signals are self-produced and secreted into the extracellular milieu. AHL molecules are produced by GNB, and AIP signals are produced by GPB [465,466]. AI-2 signaling molecules are produced by both GNB and GPB [467]. QS-mediated signal activation leads to increased virulence capabilities such as biofilm production, toxin secretion, metabolic alteration, antibiotic resistance, plasmid conjugation, and motility, making it difficult to treat bacterial infections with conventional antibiotics. QS inhibitory agents abolish signaling pathways and prevent virulence without causing selective pressures that result in drug resistance. Disrupting this bacterial communication with QS quenching agents renders pathogens more susceptible to host immune responses and antibiotics [468]. Numerous in vitro and in vivo studies have shown promising results when targeting various aspects of QS [468]. QS receptor inactivation, signal synthesis inhibition, signal degradation, and signal blockade with antibodies and combination studies with antibiotics reveal increased clearance of bacterial pathogens in various animal infection models [468]. These findings strongly imply that QS inhibitors display great therapeutic potential for treating bacterial disease. However, the clinical application of these inhibitory agents is still in its infancy. To date, data from various clinical studies show that anti-QS agents showed increased toxicity and less stability compared to their antibiotic counterparts, limiting their extensive application [200,201]. In the future, the combination of QS inhibitors and low-dose antibiotics has the potential to be used as a strategy to treat bacterial disease [469,470].

Biofilm-forming bacteria pose a major concern, as they can lead to chronic infections that cause severe public health problems and result in limited treatment options. Biofilm production is a persistent threat that increases morbidity and mortality rates, thereby imposing heavy economic pressures on worldwide healthcare systems [471]. When encased in a biofilm, bacteria become highly resistant to the administration of antibiotics, clearance by the host immune response, and are protected from mechanical removal [213,472,473]. Intact biofilms enable the spread and persistence of bacterial infections in a host. Accordingly, the development of alternative strategies and preventative methods has gained notable attention [474,475]. Currently, several anti-biofilm treatment approaches are being investigated; including (A) surface modulation of bacterial adhesion [198,312,313,476,477]; (B) disruption of bacterial cell membranes by antimicrobial peptides and lipids [478,479,480,481,482]; (C) enzyme-specific biofilm degradation [482,483,484]; (D) photodynamic approaches [485,486,487]; and (E) electrochemical mediated biofilm degradation [488,489,490]. These recent inhibitor studies have opened new prospects for controlling biofilm formation, yet although very promising, extensive research is still required to elucidate the effects of biofilm inhibitors during infection to show their suitability in a clinical setting.

Since many pathogenic bacteria elaborate toxins that lead to the manifestation and exacerbation of disease, inhibition of such secreted proteins is a promising anti-virulence treatment approach [314,491]. Exotoxins are released by many GPBs and GNBs to colonize and adapt to a changing host environment [492,493,494]. These released toxins have the capability to interact with host cells and cause direct cell damage by disrupting protective barriers or by binding to specific host cell surface receptors and initiating a signaling cascade, leading to the progression of infection and eventual death [495,496]. Furthermore, some toxins function as enzymes that thwart a mounting immune response [279,497,498]. In all cases, the targeting of secreted virulence factors has the potential to prevent the development of disease or to keep the infection from progressing [330]. To date, several active vaccines and passive immunization strategies have been developed [499]. For example, two district types of active cholera vaccines have been developed. The first consists of a live attenuated bacteria, and the second comprises an inactivated bacterial cell, which is sometimes used in combination with purified recombinant B-subunit of the cholera toxin. Additionally, passive immunization strategies with antibodies have also shown great promise [500,501,502]. As our knowledge about host-pathogen interactions expands, it is imperative that the target chosen for therapeutic purposes has a primary role as a disease-causing virulence factor. This is indeed the case with a variety of bacterial toxins that cause whopping cough, cholera, diphtheria, tetanus, botulism, and anthrax. A desirable exotoxin should provide a broad therapeutic window for prevention. As we continue to identify novel exotoxins, it should be noted that many of them are pathogen-specific and will require the development of new diagnostic tools to be effective [330].

Bacteriophage therapy was introduced into the clinic over a century ago. However, the rapid rise in multidrug resistance among many bacterial pathogens has resulted in its revival in recent years [364,368]. Phages, as bactericidal agents, have several characteristics that make them a compelling alternative to conventional antibiotics [386]. Not only are lytic phages bactericidal, but they can also increase in number over the course of treatment. They minimally disrupt the normal microflora, are effective against multidrug-resistant bacterial pathogens, and have the capability to disrupt biofilms with minimal inherent toxicity [368,377,503]. However, no matter how promising phage therapy is, challenges remain. Hurdles exist, such as compatibility with current safety and lack of regulatory standards, stability over long periods of time at varying temperatures, accessibility to infected tissue at distant anatomical regions, and potential transfer of virulence genes. Moreover, they must be screened for the presence of resistance and cytotoxicity genes. Additionally, the function of many phage genes is unknown, which may participate in unwanted or unrecognized side effects [503,504]. It should be noted that in addition to human applications, phage therapy is being explored for veterinary medicine and food product safety purposes against foodborne microorganisms such as *Listeria monocytogenes* and *Salmonella* spp. [505,506,507,508]. As we gain a greater understanding of translating phage therapy into modern-day clinical practice, phage therapy cocktails will most likely be used in the future, alone or in conjunction with lower doses of antibiotics to treat bacterial disease [382,509].

It is well established that fecal microflora transplants (FMT) are an effective treatment strategy for *Clostridioides difficile* infections (CDI). This approach restores the gut microflora diversity and functions by outcompeting the pathogen. The application of FMT is currently being investigated for the treatment of other immune-based pathologies, including allergies, inflammatory bowel disease, and other autoimmune conditions [510,511,512,513]. The dysbiosis of gut microflora often observed in these disease states provides the rationale for employing FMT as a treatment option. However, skepticism remains as to applying this technique to conditions other than CDI. A knowledge gap still exists around our understanding of the underlying mechanisms by which the gut microflora contributes to infections other than *C. difficile*. Currently, we do not have a full understanding of the ecological processes that shape the recipient’s microflora after FMT. Furthermore, microfloral signatures that can be used to personalize an FMT to identify appropriate donors or select donor-recipient pairs have not been identified and validated. Lastly, the role of adjunctive therapy, such as antibiotic treatment and nutritional guidelines, has not been established [514].

Several therapeutic modalities have been put forward, and in some cases, validation and screening studies have been performed to identify compounds targeting virulence pathways. However, apart from antibodies and toxoids, none of the anti-virulence compounds have yet to advance past Phase II clinical trials. Procedures such as phage therapy and orally delivered encapsulated FMT have not yet become common practice, but they most likely will in the near future once manufacturing and regulatory standards are established. Furthermore, the coating of medical devices with antibiofilm molecules is also likely to make it to market faster than the rest of the antimicrobial platforms mentioned above since many medical devices, such as catheters and prosthetic implants, are already being coated with alternative, regulatory-approved, compounds that are used in current clinical practice. To that end, it remains to be seen whether these new strategies live up to expectations and become employed in mainstream medicine. It is of great interest to see a greater number of published reports showing whether this new generation of antimicrobials is efficacious with a reduced development of resistance and whether they need to be used in conjunction with current antibiotics or whether will resistance render antibiotics obsolete.
